# Disaster response knowledge and its social determinants: A cross-sectional study in Beijing, China

**DOI:** 10.1371/journal.pone.0214367

**Published:** 2019-03-26

**Authors:** Tongtong Li, Qi Wang, Zheng Xie

**Affiliations:** School of Public Health, Peking University, Haidian District, Beijing, P.R.China; University of Memphis, UNITED STATES

## Abstract

**Background:**

The purpose of this study is to identify the social factors that shape the disaster response knowledge of residents in Beijing, China. This study aims to provide evidence supporting the development and promotion of effective strategies for disaster response knowledge, including natural and human-made ones.

**Methods:**

A cross-sectional household survey on disaster response knowledge was conducted in Beijing, 2015. Using the multistage sampling method, data were collected from community residents through a self-administered questionnaire, and analyzed by descriptive methods and logistic regression models.

**Results:**

Among the 847 respondents, 44.2% had advanced disaster response knowledge in general, while only 9.4% knew how to react to human-made disasters, and 61.4% had advanced natural disaster response knowledge. Rural residents and those with higher education had more disaster response knowledge (P<0.05). Economic status did not show significant effects on residents’ disaster response knowledge.

**Conclusion:**

People’s disaster response knowledge in Beijing was low, especially regarding human-made disasters. The findings implicate further health education on disaster preparedness and response should be implemented, also residents who lived in peri-urban and urban areas or with less education should be given more attention.

## Background

Disasters have always restricted economic and social development by causing widespread death and injury [1, 2]. In 2014, 13857 individuals were reported killed by disasters worldwide, and 107 million individuals were affected [3]. China is a disaster-prone countries vulnerable to hazards of both natural and human-made origin (hereafter referred to as “natural disasters” and “human-made disasters”)[4]. The 2014 World Disasters Report indicated that China accounted for 1.4‰ of total reported deaths and 5.6‰ of individuals affected by disasters in the world [3]. According to the newest data collected by China’s government, 19 million residents have been affected by natural disasters in 2016, and direct economic loss was about 50 billion Chinese Yuan [5]. Moreover, the total number of disasters is increasing, which causes crucial social damage especially considering on technological ones such as terrorist attacks [6, 7].

To mitigate the adverse effects of disasters, providing the general public with basic knowledge of how to respond to disasters (“disaster response knowledge”) is the most effective strategy [8]. This can enhance the general public’s capacity for early self-rescue and mutual-aid [9], thus reducing the damage caused to individuals and society. Developed countries often require residents to become fully aware and trained on potential threats and hazards preparedness, which is regarded as an important public health strategy for responding to unforeseen disasters [10–12]. However, little attention has been paid to disaster response knowledge in most low- and middle-income countries (e.g., China), even though these countries accounted for 51.0% of the total number of disasters globally [3]. Developing countries need to be more concerned with those residents threatened by sudden hazards [13].

Recently, the Chinese government has been trying to formulate disaster reduction plans, guidelines, and policies [14, 15]. In particular, the National Health and Family Planning Commission proposed a health literacy program in 2005, including residents’ anti-disaster awareness and skills as an important component [16–18]. In 2012, the National Health Literacy Promotion Program was established, which implemented various publicity and education activities with mass media and public speaking tours [19]. Through this program, disaster preparedness gradually entered the public consciousness. Later, the importance of health literacy was reasserted in 9^th^ Global Conference on Health Promotion (Shanghai, 2016). These initiatives represent a positive trend of the promotion of residents’ self-efficacy knowledge during disasters [20].

However, the status of residents’ disaster response knowledge under such trials remains unknown in China. A few studies have focused on disaster response knowledge at the individual level, while most have focused on special groups like students [21], teachers [22], and health-related staff [23]. Only one previous study focused on the public general was conducted in China, which showed that only 10.7% respondents had enough disaster response knowledge (2013) [24]. This percentage was undoubtedly far lower than many other countries, especially that of developed countries, where it was required that all of the residents grasped sufficient knowledge and skills to ensure self-rescue in disasters [25]. Moreover, this only study did not give a profound explanation as to why people in China lacked disaster response knowledge, and what kind of factors may affect it. Hence, this study conducted a household survey in Beijing, measuring the status of residents’ knowledge relative to preparing for a range of disasters, and exploring the social determinates affecting such preparedness.

## Methods

### Data

We conducted a health household survey in the Shunyi district of Beijing in August of 2015. This study used a multistage, stratified and random sampling design [26]. Initially, we selected two blocks in urban areas, three blocks in peri-urban areas, and four blocks in rural areas randomly according to the proportion of population in each area[3]. Within each block, we sampled two communities randomly with a consideration of their representativeness and sample size, totaling 18 communities. The local administration provided a list of all the households that had lived at each community for more than six months. Based on this list, we selected 50 households in each community by systematic sampling, with the initial subject determined by a random number. In each household, we sampled one individual randomly from those who are willing to participate in as well as able to understand/finish the survey to participate. The final sample consisted of 900 eligible households. Considering the missing data, this study enrolled 847 in the analysis with a response rate of 96.3%.

A structured questionnaire was designed by our team based on the research experience of preventive medicine and health management, with consideration of the relevant literature [27, 28]. After two rounds of discussion among the team and three rounds of discussion with external experts, the final version of the questionnaire was constructed of three sections. The first section included demographic information such as age, gender, educational level, household income, and family members. The second section contained 34 multiple-choice questions as a test about knowledge of potential threats and hazards preparedness. Before the survey, reliability and validation of the questionnaire was conformed using a pilot test and investigators were trained to ensure consistency. The participants were then asked to finish the questionnaire independently. All the survey was in Chinese, which was their native language.

### Ethics approval

Ethics approval for this study was obtained from Peking University Institutional Review Board (protocol number IRB00001052-16018). All the respondents in this study had signed statements on consent to participate.

### Measures

Total disaster response knowledge was measured by 34 items, including the knowledge of how to reflect in earthquakes, wildfires, floods, respiratory and digestive infectious diseases, transportation and industrial accidents, and so on. For each item, we gave a value of 1 for a right answer and 0 for a wrong answer. Thus, the sum score ranged from 0 to 34. Because these items constitute essential knowledge for potential threats and hazards preparedness, we considered residents who scored more than 27, with 80% right, to possess advanced disaster response knowledge, while those with scores of 0–27 as having basic knowledge.

To show the status of residents’ disaster response knowledge in detail, we followed the World Disaster Report and divided these 34 items into two categories: natural disaster response knowledge and human-made disasters response knowledge [3]. According to this category method, 29 items belonged to natural disasters response knowledge, and 5 items belonged to human-made disasters response knowledge. Using the same measure with the total score, we considered residents who scored more than 23 (with 80% right) to possess advanced natural disaster response knowledge, while those with scores of 0–23 were considered as having basic natural disaster response knowledge. Similarly, residents who scored above or equal to 4 were regarded as possessors of advanced human-made disasters knowledge.

Control variables included age (0–49, 50–59, and 60 or over), gender, and education level (completion of primary school or less, completion of junior high school, and completion of secondary school or higher). We used per capita household income as a proxy for expected socioeconomic level to adjust for family size, which we assessed with the questions “How much money did your household earn yearly on average?” and “How many family members in your household?” Then, we created three levels of per capita income from the responses: 0–1500 *yuan* per capita a year (low), 1501–3000 *yuan* per capita a year (ordinary), and more than 3000 *yuan* per capita a year (high).

### Analyses

We conducted the analyses using SPSS version 12.0. Among these, we used descriptive statistics to describe the characteristics of the study population and the status of residents’ different kinds of disaster response knowledge. To determine the differences in individual variables according to total disaster response knowledge, we also performed Chi-squared tests. Using logistic regression, we fitted the relative data with residents’ total disaster response knowledge status as the outcome, controlling by the social determinants (age, gender, education, income status, and household location). Odds ratios (ORs) and their 95% confidence intervals (CIs) for each independent variable were analyzed. Moreover, weighted proportions accounting for sampling design were considered in all analyses.

## Results

### Descriptive statistics of the sample

Table 1 shows that 489 (57.7%) participants were female and 345 (42.0%) were no younger than 60 years old. More than 40% of the participants held a junior high school degree (46.1%), and lived in rural areas (65.5%).

**Table 1:** Descriptive statistics.

Variable	Number	Percentage (%)^#^
Gender		
Male	358	42.3
Female	489	57.7
Age (years)		
0–49	245	26.5
50–59	257	31.5
60 +	345	42.0
Education		
≤Primary school	183	23.3
Junior high school	380	46.1
≥Secondary school	284	30.6
Per capita household income		
Low	217	29.5
Ordinary	285	33.2
High	345	37.3
Household location		
Rural	386	65.5
Peri-urban	288	16.8
Urban	173	17.8

^#^ Weighted proportions accounting for sampling design were used to calculate proportions.

### Disaster response knowledge distribution

**Fig 1:**
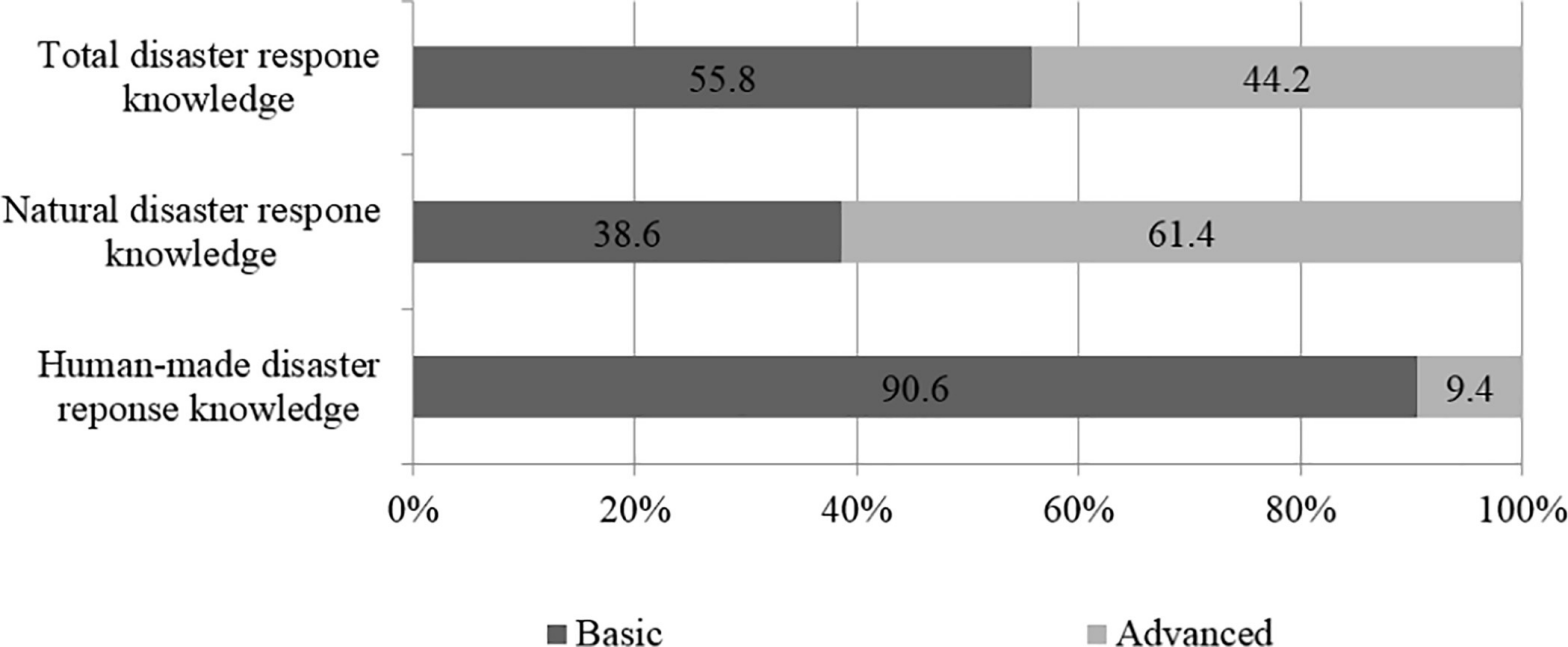
Status of residents' disaster response knowledge in Beijing, China.

In general, 44.2% of participants had advanced disaster response knowledge (Fig 1). Meantime, 61.4% participants answered they knew how to deal with natural disasters. However, only 9.4% of participants knew how to respond to human-made disasters, when we divided disasters into different forms.

### Differences in residents’ disaster response knowledge

Among 847 participants, those who were 0–49 years old, or lived in rural areas were more likely to have advanced disaster response knowledge (P<0.001). Furthermore, the advanced group had higher level of education (P<0.001) (Table 2).

**Table 2:** The differences of residents’ characteristics by disaster response knowledge.

Variables	Basic (%)^#^	Advanced (%)^#^	P value ^a^
Gender			< 0.001
Male	194 (38.7)	164 (46.9)	
Female	295(61.3)	194 (53.1)	
Age (years)			< 0.001
0–49	125 (24.0)	120 (29.6)	
50–59	154 (32.5)	103 (30.2)	
60 +	210 (43.5)	135 (40.2)	
Education			< 0.001
≤Primary school	123 (25.7)	60 (20.2)	
Junior high school	230 (47.5)	150 (44.4)	
≥Secondary school	136 (26.8)	148 (35.4)	
Per capita household income			0.027
Low	119 (28.3)	98 (30.8)	
Ordinary	170 (34.0)	115 (32.3)	
High	200 (37.7)	145 (36.9)	
Household location			< 0.001
Rural	207 (62.0)	179 (69.8)	
Peri-urban	188 (19.7)	100 (13.1)	
Urban	94 (18.3)	79 (17.1)	

^a^ P value by chi-square test.

^#^ Weighted proportions accounting for sampling design were used to calculate proportions.

### Logistic regression estimates of residents’ total disaster response knowledge

Table 3 shows the results of logistic regression analysis models testing relative factors associated with residents’ disaster response knowledge. The participants with more education were more likely to possess advanced disaster response knowledge (OR = 1.181, 95%CI: 1.053–1.324; OR = 1.936, 95%CI: 1.685–2.224). Compared with residents in rural areas, those in peri-urban or urban areas knew less about how to respond to disasters (OR = 0.619, 95%CI: 0.544–0.704; OR = 0.564, 95%CI: 0.502–0.635). Simultaneously, economic status did not show significant effects on residents’ disaster response knowledge.

**Table 3:** Logistic regression estimates of residents' disaster response knowledge ^#^.

Variables	OR	95% C.I.
Gender		
Male (ref)		
Female	0.709**	0.650,0.772
Age (years)		
0–49 (ref)		
50–59	0.778**	0.695,0.871
60 +	0.892	0.793,1.002
Education		
≤Primary school (ref)		
Junior high school	1.181*	1.053,1.324
≥Secondary school	1.936**	1.685,2.224
Per capita household income		
Low (ref)		
Average	0.912	0.820,1.014
High	0.936	0.839,1.044
Household location		
Rural (ref)		
Peri-urban	0.619**	0.544,0.704
Urban	0.564**	0.502,0.635

*P<0.050

**P<0.001.

^#^ Weighted proportions accounting for sampling design were used in this regression.

## Discussion

The survey showed that for Beijing in 2015 the residents’ percentage of possessing advanced disaster response knowledge was 44.2%, higher than in the previous study in China [24]. This may indicate a recent increase, and a positive trend, for Chinese residents’ disaster response knowledge, although the baseline situation may vary across different cities [9]. However, 44.2% is still lower than the target of national health literacy program, which aims a universe coverage of disaster response knowledge. This may be due to the sustainability and availability of reinforcing activities. For instance, Japan is also a disaster-prone country, but their disaster response knowledge coverage is near 100% [29], far higher than China. The difference between these two countries is that Japan has implemented annual education programs regularly [30] and even incorporated disaster self-efficacy education into their school education, ensuring benefits for all residents [31]. However, such strategy has not been effectively applied in China. It may, thus, imply that more sustainable and available educational activities for the general public should be implemented, to promote knowledge and increase capacity for self-rescue and mutual-aid during disasters.

Compared to individuals who live in rural areas, those who live in peri-urban/ urban areas are less advantaged regarding disaster response knowledge. This deficit may be explained by empirical studies, which have claimed that location-based disaster response knowledge is related to the geographic distribution of disasters [32, 33]. Most rural areas in Shunyi District are around by mountain and rivers with higher risk to suffer disasters than urban and peri-urban areas. Thus, residents who live in rural areas would prepare more sufficiently to against disasters. However, it is not a convincing excuse that peri-urban or urban residents do not need equal accumulation of disaster response knowledge. Therefore, our results implied that more attention should be given to peri-urban or urban residents on health education, especially disaster response knowledge in the future.

The results showed that economic status had no significant effects on residents’ disaster response knowledge, showing health equality in this area of disaster control. Residents who had better economic status did not show any advantage over those who earned less, suggesting that residents should be given the same attention on disaster education no matter what kinds of economic status they had. This result implied that our promotion strategies should reach the entire target population without income bias [34].

Unlike prior studies that held gender as a control variable in disaster response knowledge analyses [35, 36], we believes that gender is one key factor that should not be ignored, at least not in China [8]. Indeed, gender inequality in health remains a pervasive factor in many related studies [37]. Our study showed that males displayed better accumulation of disaster response knowledge than females, implying that gender inequality also exited on disaster control.

Our results show that education level has the strongest correlation with residents’ disaster response knowledge. Specifically, those participants with higher education are more likely to have advanced disaster response knowledge, in accordance with previous studies in the same field [38, 39]. This is may be because individuals who received higher education are easier to receive or feel interested in disaster response knowledge. Fortunately, the Chinese education system has changed since 1949, increasing the university/college enrollment rate dramatically [40]. This increase suggests a positive trend towards the long-term promotion of disaster response knowledge, as with other education-related health issues in China [41].

The findings suggest that fewer participants know how to react in human-made disasters than in natural disasters. This difference may be partly due to the interpersonal nature of human-made disasters compared to natural disasters [42]. Natural disasters often have unique and clear prevention strategies, while the individual preparedness for human-made disasters is more detailed and complex [43]. Thus, it is more difficult to realize publicity and education for human-made disasters than for natural disasters, leaving to a relatively negative result.

There are several limitations in this study. Firstly, the cross-sectional nature of the study dictates that only correlation can be studied, and not causation. Secondly, due to cultural and geography differences, there is lack of an acknowledged and unified survey scale on residents’ disaster response knowledge measuring worldwide. Thus precise comparisons between our study and previous ones are limited considering varied disaster response knowledge measuring methods, although such an analysis can help identify a rough trend. Meanwhile, our results may also affected by varied measuring methods. Therefore, further study using other measuring methods is needed to help confirm our findings. Thirdly, only one district in Beijing was sampled, potentially jeopardizing the external validity and generalizability of the results. Further research in different parts of Beijing, and in China, is needed to attain a better and more thorough understanding of the situation of residents’ disaster response knowledge and its social determinants.

## Conclusion

In general, only 42.3% residents had advanced disaster response knowledge for Beijing in 2015, showing a positive trend but still needed to be promoted in the long-term. Residents who lived in peri-urban/urban areas or with less education had insufficient disaster response knowledge. More disaster education or other kinds of policy support should be given on these vulnerable groups.

## Supporting information

S1 File Questionnaire in English. (PDF)

S2 File Questionnaire in Chinese. (PDF)

S3 File Anonymized data set. (SAV)

## Abbreviations

ORs: Odds ratios
Cis: confidence intervals
