# Supplementary material for: Disaster response knowledge and its social determinants: A cross-sectional study in Beijing, China
Source: PLoS One. 2019 Mar 26;14(3):e0214367. doi: 10.1371/journal.pone.0214367 (PMC6435165; doi:10.1371/journal.pone.0214367)
Supplement: S1 File — (PDF) [file pone.0214367.s001.pdf]

Questionnaire

1. Do you think the following methods can effectively prevent respiratory infectious diseases?

(1) No spitting ①Yes ②No ③Do not know

(2) Don't drink unboiled water ①Yes ②No ③Do not know

(3) Keep ventilated ①Yes ②No ③Do not know

(4) Do not go to crowded places in the disease prevalent seasons

①Yes ②No ③Do not know

2. Do you think the following methods can effectively prevent digestive tract infections?

(1) Wear a mask ①Yes ②No ③Do not know

(2) Regular hand-washing ①Yes ②No ③Do not know

(3) Raw and cooked foods are stored and processed separately

①Yes ②No ③Do not know

(4) Do not pooh on the streets ①Yes ②No ③Do not know

3. If a lot of sick and dead livestock are found around you, is the following behavior correct?

(1) Kill and cook thoroughly before eating ①Yes ②No ③Do not know

(2) General method burial ①Yes ②No ③Do not know

(3) Burn and bury deep ①Yes ②No ③Do not know

(4) Report to animal quarantine ①Yes ②No ③Do not know

4. If someone accidentally eats pesticides or other toxic chemicals, it is correct to do the following:

① Drink plenty of water immediately and seek medical advice

② Immediately stimulate the root of the tongue after emetic treatment

③ Drink soap and water immediately before seeking medical advice

④ Do not know

5. If nausea, vomiting, abdominal pain and diarrhea are found in many people after a meal, should all food eaten recently be destroyed immediately?

①Yes ②No ③Do not know

6. If there is a serious traffic accident, which of the following telephone Numbers can be called for help?

①112 ②114 ③122 ④Do not know

7. If you are in the location of a gas attack or chemical leakage, please choose the right direction of escape:

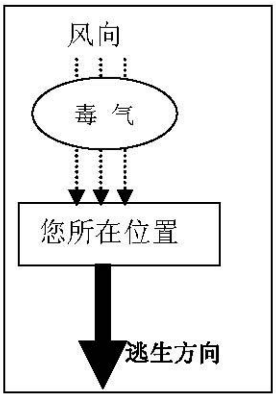

①

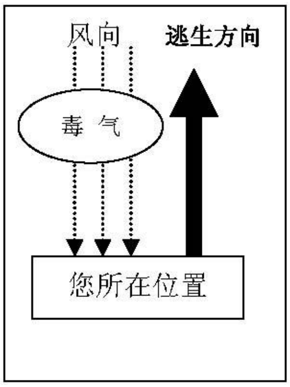

②

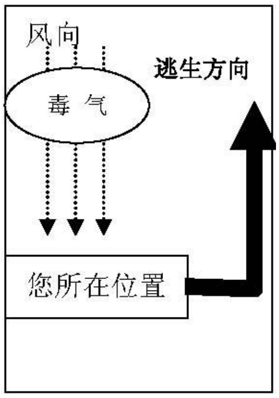

③

④Do not know

8. If there is a radioactive accident nearby, you think the following is correct:

① Keep ventilated

② Hide under trees or near tall building

③ Immediately hide inside and close doors and windows

④ Run outside and wait for help

⑤ Do not know

9. If you are indoors during an earthquake, what you think is the right thing to do:

① Lie flat on your back

② Stretch and relax as much as you can

③ Curl up as close as you can and put your hands on your head

④ Keep your body standing

⑤ Do not know

10. If you are outdoors during an earthquake, what you think is the right thing to do:

① Return to a room immediately

② Choose to squat or lie down in an open area

③ Hide near tall buildings

④ Hide near tall trees

⑤ Do not know

11. In an earthquake, if there is no time to get out of the house, the indoor unsafe place is:

① Narrow space in toilet, kitchen, storeroom narrow space

② Next to solid furniture

③ Internal load-bearing wall corner

④ On a window or balcony

⑤ Do not know

12. Is the following about the earthquake self-help and mutual rescue correct?

(1) Once buried, must insist on calling for help loudly

①Yes ②No ③Do not know

(2) Once a buried person has been rescued, he/she should eat a large amount of food and water immediately

①Yes ②No ③Do not know

(3) If the buried person can not be rescued at once, his head should be exposed to remove the dust from the mouth and nose

①Yes ②No ③Do not know

(4) If you break water for a long time in an earthquake, you can save your own urine and quench your thirst

①Yes ②No ③Do not know

13． Do you think the following measures for escaping from the flood are correct？

(1) Run to higher ground for a while ①Yes ②No ③Do not know

(2) Hold on to something as fixed as possible ①Yes ②No ③Do not know

(3) Hold on to floating objects as much as possible ①Yes ②No ③Do not know

(4) Climb the wire tower ①Yes ②No ③Do not know

(5) Try to breathe through your mouth when drowning

①Yes ②No ③Do not know

14． Do you think the following measures for escaping from a fire are correct?

(1) If a tall building catches fire, take an elevator to evacuate

①Yes ②No ③Do not know

(2) If smoke fills the room during a fire, cover your nose and mouth with a wet towel and leave□

①Yes ②No ③Do not know

(3) When there is no way out, call for help from a window or balcony

①Yes ②No ③Do not know

(4) Close the door when you leave the room

①Yes ②No ③Do not know

(5) If there is a fire downstairs, run upstairs

①Yes ②No ③Do not know

Personal Information:

1. Gender： ①Male ②Female
2. Year of Birth：
3. How many family members in your household?
4. How much money did your household earn yearly on average?

5. Education：
   ① None
   ② Primary school
   ③ Junior high school
   ④ Senior high school
   ⑤ Technician training school
   ⑥ Technical secondary school (technical secondary school)
   ⑦ Junior college
   ⑧ Bachelor degree or above
6. Household location： ①Urban ②Rural ③Peri-urban
