# Supplementary material for: Disaster response knowledge and its social determinants: A cross-sectional study in Beijing, China
Source: PLoS One. 2019 Mar 26;14(3):e0214367. doi: 10.1371/journal.pone.0214367 (PMC6435165; doi:10.1371/journal.pone.0214367)
Supplement: S2 File — (PDF) [file pone.0214367.s002.pdf]

卫生应急相关知识调查问卷

1．您认为以下方法能够有效预防呼吸道传染病吗？（请分别判断）

（1）不随地吐痰 □①能 □②不能 □③不知道

（2）不喝生水 □①能 □②不能 □③不知道

（3）勤通风 □①能 □②不能 □③不知道

（4）流行季节不到人群密集的场所 □①能 □②不能 □③不知道

2．您认为以下方法能够有效预防消化道传染病吗？（请分别判断）

（1）戴口罩 □①能 □②不能 □③不知道

（2）勤洗手 □①能 □②不能 □③不知道

（3）生熟食物分开保存和加工 □①能 □②不能 □③不知道

（4）不随地大小便 □①能 □②不能 □③不知道

3．如果您周围发现大量的病死禽畜，以下做法正确吗？

（1）宰杀，彻底煮熟后食用 □①正确 □②不正确 □③不知道

（2）一般方法掩埋 □①正确 □②不正确 □③不知道

（3）焚烧后深埋 □①正确 □②不正确 □③不知道

（4）报告动物检疫部门 □①正确 □②不正确 □③不知道

4．如果有人误食了农药或其他有毒化学品，以下做法中正确的是：

□①立即喝大量清水后就医 □②立即刺激舌根催吐后就医

□③立即喝肥皂水后就医 □④不知道

5．聚餐后，如果发现有很多人出现恶心、呕吐、腹痛、腹泻的症状，应该立即销毁最近吃过的所有食物，这种做法正确吗？

□①正确 □②不正确 □③不知道

6．如果发生了重大的交通事故，可以拨打以下哪个电话求助？

□①112 □②114 □③122 □④不知道

7．如果您所在的位置遭毒气袭击或化学品泄漏，请选择正确的逃生方向：

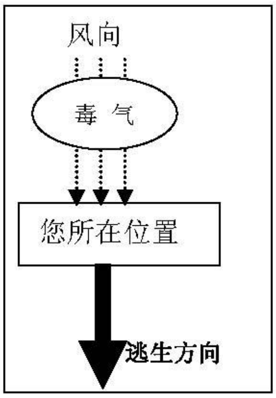

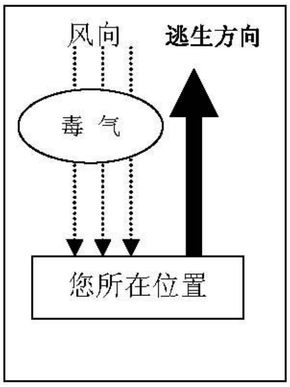

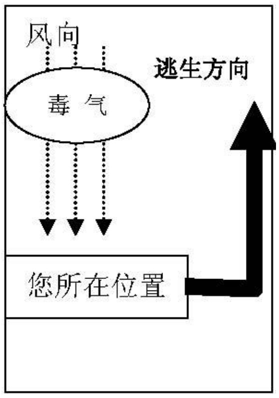

□① □② □③ □④不知道

8．如果周围有放射性事故发生，您认为以下做法正确的是：

□①打开门窗，保持通风 □②躲到树下或高大建筑物旁边

□③立即躲避到屋内，关闭门窗 □④跑到室外空地等待救援 □⑤不知道

9．下列室内躲避地震的做法中，您认为正确的是：

□①平躺在室内的空地上 □②尽可能舒展身体，保持放松

□③身体尽量蜷曲缩小，双手抱头 □④保持身体站立状态 □⑤不知道

10．地震时，如果您在室外，您认为正确的做法是：

□①立即返回室内　　　　　　□②就地选择开阔地蹲下或趴下
□③躲到高大建筑物附近　　　□④跑到大树底下　　　□⑤不知道

11．地震时，若来不及逃出屋外，室内不安全的地点是：
□①卫生间、厨房、储藏室等狭小空间　　　　　　　□②坚固的家具旁边
□③内部的承重墙墙角　　　□④窗户边或阳台上　　　□⑤不知道

12．以下关于地震中自救互救的做法正确吗？（请分别判断）
(1) 地震时如果被埋压，一定要坚持长时间大声呼救
□①正确　　□②不正确　　□③不知道
(2) 救出地震中被埋压的人员后应立即喂以大量食物和水
□①正确　　□②不正确　　□③不知道
(3) 一时无法救出被埋压者，应设法先暴露其头部清除口鼻尘土
□①正确　　□②不正确　　□③不知道
(4) 地震中如果长时间断水断粮，可以保存自己的尿液解渴
□①正确　　□②不正确　　□③不知道

13．以下关于洪水中逃生的做法，您认为正确吗？（请分别判断）

(1) 跑到高处暂时躲避	□①正确	□②不正确	□③不知道
(2) 尽可能抓住固定的东西	□①正确	□②不正确	□③不知道
(3) 尽可能抓住漂浮物体	□①正确	□②不正确	□③不知道
(4) 爬上电线铁塔	□①正确	□②不正确	□③不知道
(5) 一旦溺水应尽量用嘴呼吸	□①正确	□②不正确	□③不知道

14．以下关于火灾中逃生的做法，您认为正确吗？（请分别判断）

(1) 如果高楼失火，可以乘电梯撤离	□①正确	□②不正确	□③不知道
(2) 若火灾时房内弥漫浓烟，用湿毛巾捂住口鼻离开	□①正确	□②不正确	□③不知道
(3) 无路可逃时，靠近窗户或阳台呼救	□①正确	□②不正确	□③不知道
(4) 火灾逃生离开房间时关闭房门	□①正确	□②不正确	□③不知道
(5) 如果楼下发生火灾，往楼上跑	□①正确	□②不正确	□③不知道

一般信息

1. 性别：　⑴男　　⑵女
2. 出生日期：　年份　（填写4位数字，如：1998）
3. 户籍人口中，近6个月内有几人在家里居住？
4. 您家前一年总收入约为多少元？（城镇居民家庭为可支配收入，农村居民家庭为纯收入）
5. 文化程度：　⑴没上过学　⑵小学　⑶初中
　⑷高中　⑸技工学校　⑹中专(中技)
　⑺大专　⑻本科及以上
6. 家庭住址：　⑴城市　⑵农村　⑶城镇
